# NMR Methods to Study Dynamic Allostery

**DOI:** 10.1371/journal.pcbi.1004620

**Published:** 2016-03-10

**Authors:** Sarina Grutsch, Sven Brüschweiler, Martin Tollinger

**Affiliations:** 1 Institute of Organic Chemistry, Center for Molecular Biosciences Innsbruck (CMBI), University of Innsbruck, Innsbruck, Austria; 2 Department of Computational & Structural Biology, Max F. Perutz Laboratories, Campus Vienna Biocenter 5, Vienna, Austria; University of North Texas System College of Pharmacy, UNITED STATES

## Abstract

Nuclear magnetic resonance (NMR) spectroscopy provides a unique toolbox of experimental probes for studying dynamic processes on a wide range of timescales, ranging from picoseconds to milliseconds and beyond. Along with NMR hardware developments, recent methodological advancements have enabled the characterization of allosteric proteins at unprecedented detail, revealing intriguing aspects of allosteric mechanisms and increasing the proportion of the conformational ensemble that can be observed by experiment. Here, we present an overview of NMR spectroscopic methods for characterizing equilibrium fluctuations in free and bound states of allosteric proteins that have been most influential in the field. By combining NMR experimental approaches with molecular simulations, atomistic-level descriptions of the mechanisms by which allosteric phenomena take place are now within reach.

## Introduction

In allosteric proteins, information about binding events is communicated between remote sites that are linked by a network of interactions. This allosteric communication process typically involves a specific redistribution of the accessible conformational states and can, but does not have to, produce an experimentally observable structural rearrangement [1–3]. In either case, allosteric communication is fundamentally dynamic in nature [4–8] and can be experimentally studied in a quantitative manner by nuclear magnetic resonance (NMR) spectroscopy. It is of particular interest to identify and characterize equilibrium dynamics of allosteric proteins, i.e., conformational fluctuations that are permanently present under equilibrium conditions. Long-range fluctuations can involve networks of protein sites spanning distances up to 20 Å and more, and their modulation in response to specific binding of a ligand molecule can be essential for allosteric communication between remote binding sites.

The last decade has witnessed the development and optimization of exciting new NMR spectroscopic methods to study such equilibrium dynamics in the free and bound states of biomolecules in atomistic detail. The experimental characterization of such processes in proteins and (ribo)nucleic acids is now feasible for timescales ranging from picoseconds to seconds (and slower), at atomic resolution and in a quantitative manner [9,10]. While pico- to nanosecond timescale equilibrium dynamics are commonly studied by combined measurement of diverse spin relaxation parameters pertaining to NMR-active nuclei, processes occurring in microseconds and milliseconds are accessible through experiments that are sensitive to the presence of exchange line broadening in NMR spectra. Among these, relaxation dispersion techniques are particularly useful to characterize conformational heterogeneity and transitions between different conformational states, including high-energy states that are populated to very low degrees. Dynamic processes can also be studied by residual dipolar couplings, magnetization transfer techniques, or real-time observation.

With respect to allosteric proteins, application of these NMR techniques has revealed subtle details of dynamic coupling mechanisms. Most strikingly, the focus of interest has steadily been shifting from very fast (pico- to nanosecond) processes toward the micro- to millisecond time regime and beyond. This has been afforded, on the one hand, by methodological advancements, which now facilitate rapid and efficient acquisition of relaxation dispersion data and the implementation of this technique as a standard experimental tool in most NMR laboratories. On the other hand, the development of new procedures for site-specific isotope labeling has prompted the application of NMR spectroscopy to proteins and protein complexes of high-molecular weight that had not been amenable to investigation before. Because NMR experiments by nature yield quantitative information, these data provide extremely valuable parameters for computational chemists. It is, thus, not surprising that computer simulation techniques have been applied to slower timescale allosteric processes in the recent past using enhanced sampling protocols. Together, simulation and experiment have provided exciting insights into intricate molecular mechanisms of allosteric proteins.

**Fig 1:**
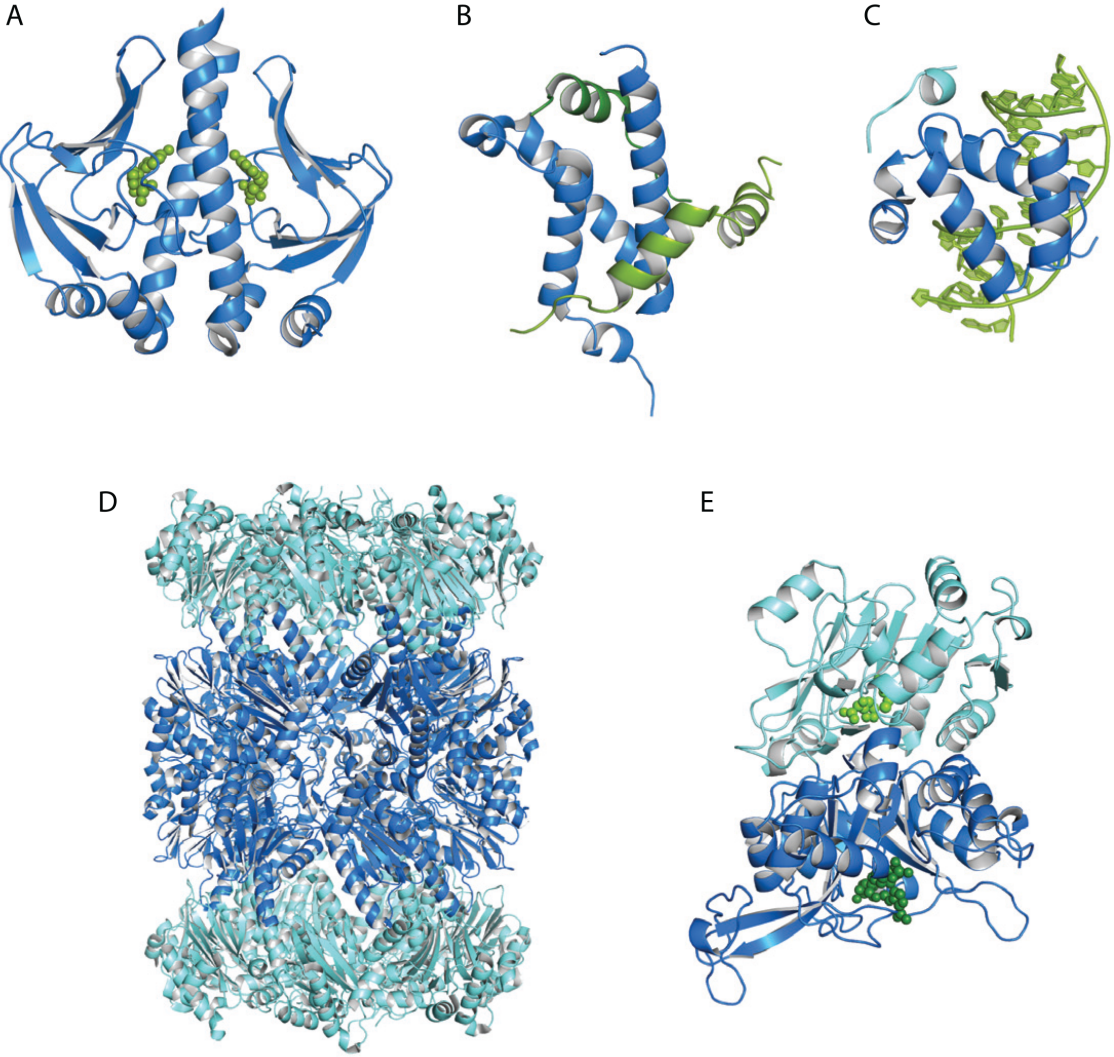
Three-dimensional structures of allosteric proteins. (A) The homodimeric catabolite activator protein (CAP) bound to two molecules of cAMP (green spheres; Protein Data Bank [PDB] identifier 1G6N) [11]. (B) The KIX domain of CREB-binding protein (CBP; blue) in complex with the peptides mixed-lineage leukemia (MLL; top, dark green, residues 2,840−2,858) and phosphorylated kinase-inducible domain (pKID; light green, residues 116−149; PDB identifier 2LXT) [12]. (C) The PBX1 homeodomain (PBX-HD, blue) bound to DNA (green) and the HoxB1 homeodomain peptide (light blue, residues 177−185; PDB identifier 1B72) [13]. (D) The 20*S* core particle proteasome (20*S* CP); α- and β-subunits are shown in light and dark blue, respectively (PDB identifier 3C91) [14]. (E) The heterodimeric enzyme imidazole glycerol phosphate synthase (IGPS), subunits HisH (light blue) and HisF (dark blue). The allosteric effector PRFAR (dark green spheres) and the substrate glutamine (light green spheres) are shown (PDB identifier 1OX5) [15]. Prepared using PyMOL (The PyMOL Molecular Graphics System, Version 1.41, Schrödinger LLC).

In this review, we provide an overview of the most commonly used NMR spectroscopic techniques to study dynamic allosteric phenomena, along with recent methodological developments. These experimental approaches are illustrated using five representative proteins and protein complexes [11–15], for which the allosteric mechanisms have been characterized in detail (Fig 1).

## Relaxation Dispersion Experiments

**Fig 2:**
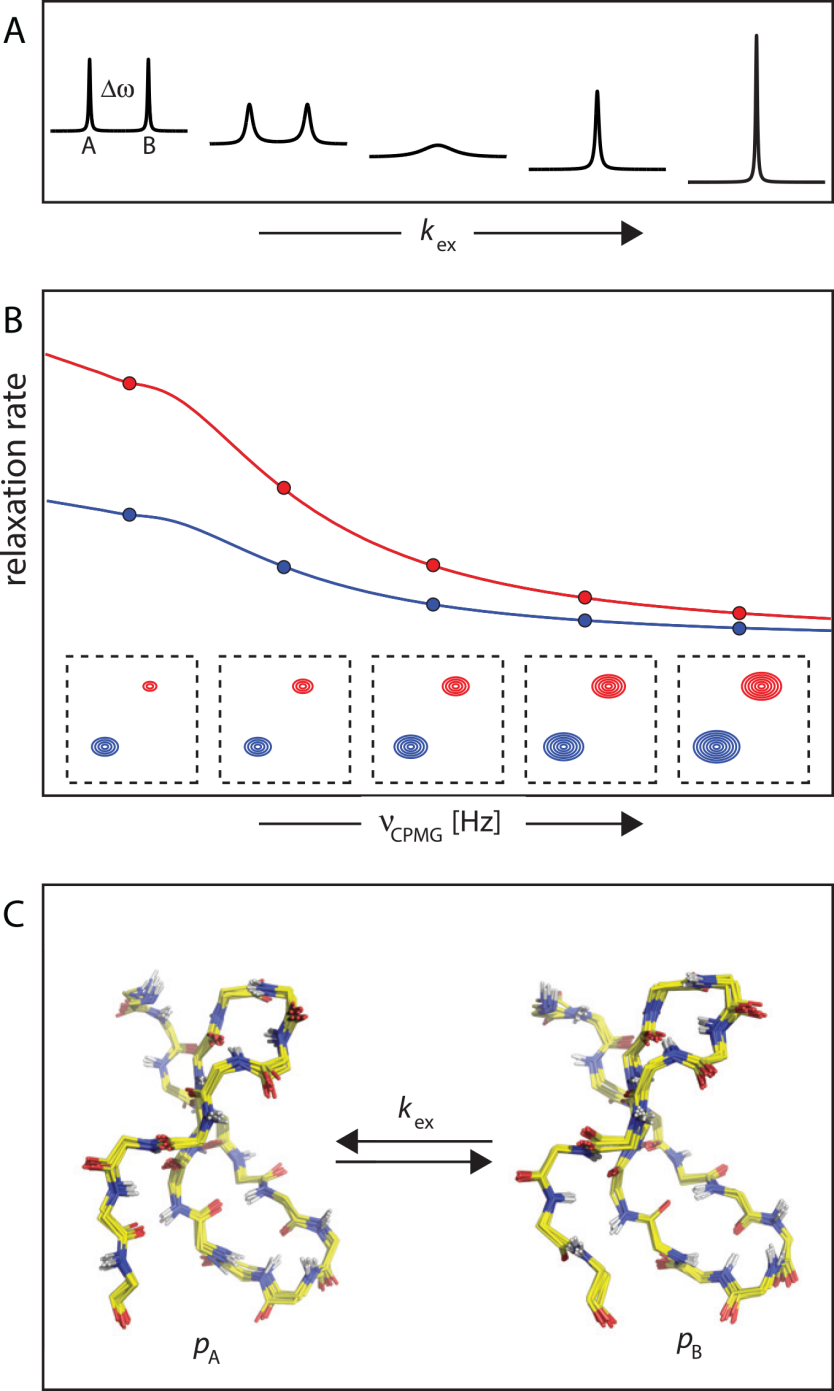
Relaxation dispersion experiments. (A) Transitions (exchange) between two states, A and B, causes line broadening of resonances in NMR spectra if the chemical shifts of the two states are different (Δω ≠ 0) and the exchange rate constant, *k*_ex_, is in the micro- to millisecond time range. (B) In the typical experimental setup for CPMG relaxation dispersion measurements, resonance intensities at multiple protein sites (e.g., all backbone amide NH groups) are measured at variable CPMG frequencies (bottom). Relaxation dispersion profiles are obtained by converting these intensities to transverse relaxation rates (top). (C) Analysis of RD profiles yields information on kinetic (*k*_ex_), thermodynamic (fractional populations *p*_A_, *p*_B_), and structural (Δω) parameters of the underlying dynamic exchange process(es). RD experiments provide this information only for protein sites with different local structures in states A and B (Δω ≠ 0).

Dynamic processes occurring on a timescale of micro- to milliseconds can contribute to the line-widths of resonances in NMR spectra through exchange line broadening, provided that the chemical shifts of the involved states are different (Fig 2). NMR relaxation dispersion (RD) techniques are excellent experimental tools to probe such processes quantitatively and at atomic resolution [10]. In these experiments, relaxation rate constants are measured under the effect of an adjustable radio frequency field, which is typically achieved by application of a series of refocusing pulses with variable pulse spacing (Carr-Purcell-Meiboom-Gill [CPMG] experiments) or using a continuous radio frequency field [16]. In either case, relaxation dispersion profiles are obtained, which are characteristic for the kinetic, thermodynamic, and structural features of the underlying dynamic process. RD experiments provide information about micro- to millisecond dynamic processes so long as the involved states are populated to more than ~0.5%, corresponding Δ*G* more than ~3 kcal/mol at 25°C.

From an experimental perspective, interference due to scalar couplings between adjacent NMR-active nuclei can result in contributions to RD profiles that are not related to dynamics [17]. Standard RD experiments thus pertain to nuclei that (i) are effectively "isolated" (scalar couplings less than ~1 Hz) from each other, such as backbone amide ^15^N, and (ii) can be isotope-labeled by bacterial expression without requiring specific labeling schemes. CPMG-based RD experiments have also been devised for ^1^H and ^13^C nuclei in the protein backbone, in protein side chains, along with relaxation-optimized experiments for probing high-molecular weight proteins [10]. In many of these experiments, scalar couplings are either suppressed experimentally or eliminated by use of specifically isotope-labeled protein samples [18]. With the exception of deuteration, which modulates the strengths of van der Waal's interactions and can, thus, have an effect on structural dynamics, isotope labeling is generally considered noninvasive. For (ribo)nucleic acids, due to significant resonance overlap in NMR spectra and the fairly complex scalar coupling network, site-specific isotope labeling plays a major role in developing CPMG-based RD experimental schemes [19].

Experimental RD data are analyzed by fitting exact [20] or approximative analytical equations that are available for certain timescales [10] or by use of numerical solutions of the Bloch equations, including magnetization exchange effects [21]. For a simple two-state exchanging system A < = > B, analysis of RD data yields information on the kinetics of the underlying dynamic process in terms of the exchange rate constant, *k*_ex_, the chemical shift difference between the states, and their fractional populations. Temperature-dependent experiments enable the quantification of thermodynamic parameters Δ*H*, Δ*S*, and activation-free energies *E*_a_ by standard van 't Hoff and Arrhenius analysis. In practice, RD profiles of multiple "reporters" (i.e., nuclei) recorded at two or more static magnetic field strengths are fit together to obtain an accurate description of the underlying dynamic process(es) [22]. Data analysis typically involves a collective motional model, assuming a common exchange rate constant and fractional populations but nucleus-specific chemical shift differences between states. By this approach, collective motions (in which all nuclei sense the same kinetics) can be identified while outliers become evident [23].

Relaxation dispersion NMR techniques have been employed to examine the allosteric mechanisms of various proteins and protein complexes with molecular weights up to 230 kDa [24–29]. As an example, for the catabolite activator protein (CAP, Fig 1A), RD studies revealed the dynamic process through which two ligand binding sites that are more than 24 Å apart mediate allostery [30]. CAP is a homodimeric protein in which each subunit contains a ligand (cAMP) binding site at its N-terminus and a DNA-binding domain at its C-terminus. While the two cAMP molecules bind to CAP at distinct sites, binding of the first molecule significantly reduces the affinity for the second cAMP molecule. Using a comprehensive set of NMR relaxation dispersion experiments of apo-CAP along with singly and doubly liganded forms of the protein, it was shown that binding of the first cAMP ligand significantly enhances micro- to millisecond dynamics of CAP of almost all amino acids in both subunits, thereby linking the two binding sites. Subsequent binding of the second cAMP molecule is accompanied by substantial rigidification of the entire protein backbone and a near complete loss of dynamics, further highlighting the role of conformational entropy for allosteric regulation in CAP. In addition, RD data of different variants of CAP were used to characterize the regulation of the DNA-binding domain, which is stimulated by cAMP [31], and for analyzing the role of sparsely populated ("excited") states in allosteric inhibition [32]. In a recent computational study using coarse-grained elastic network models (ENMs) of CAP, low-frequency correlated motions throughout the protein were found to be affected by binding both the first and the second molecules of cAMP, suggesting that allostery is indeed mediated by equilibrium fluctuations [33]. All-atom molecular dynamics (MD) simulations of micro- to millisecond dynamics in CAP are not available to date.

Allosteric coupling is notably different for the KIX domain of CREB-binding protein (CBP), a three-helix bundle protein that physically interlinks transcription factors via the formation of a ternary complex (Fig 1B). In KIX, ligand peptides are bound to remote binding sites, yet binding of either ligand mutually enhances the affinity for the second ligand. NMR relaxation dispersion experiments showed that allosteric communication proceeds through a defined redistribution of accessible conformational states [34]. Using backbone and side-chain RD experiments, it was shown that KIX bound by only one ligand peptide (mixed-lineage leukemia [MLL]) is conformationally heterogeneous in solution. Indeed, in this binary complex, an "excited" state is populated (to 7% ± 0.3% at 25°C) in which KIX structurally resembles the ternary complex. Thus, even in the absence of an interaction partner for the second binding site, the remote binding surface appears to be partially preformed. NMR titration experiments further imply that binding of the second ligand is accomplished through a redistribution of states to form the ternary complex. Moreover, the RD data showed that allosteric coupling proceeds through a network of residues that bridge the two binding sites in KIX at a rate *k*_ex_ of 330 ± 40s^-1^, including part of the hydrophobic core. The allosteric communication pathway in KIX was recently examined in atomistic detail by combining all-atom molecular dynamics with enhanced sampling techniques [35]. Using well-tempered ensemble metadynamics to probe conformational states with millisecond lifetimes, the presence of an excited state in the binary complex of KIX with MLL was verified, and its structural similarity to the ternary complex (rather than the binary complex) was confirmed. Of note, this molecular simulation study pointed out the critical role of side-chain dihedral angle variations of amino acid residues in the KIX hydrophobic core as a means for transmitting allosteric information between binding sites.

Relaxation dispersion experiments are not restricted to simple two-state processes. However, the analysis of RD data becomes increasingly complex with increasing numbers of exchanging states and, hence, the number of adjustable parameters in the fitting procedure. Characterization of processes that involve three interconverting states requires RD measurement of different single-quantum and multiple-quantum (MQ) coherences [36], controlled perturbation of the equilibrium through temperature variation or addition of ligand [37], or orthogonal NMR experiments to constrain adjustable parameters (e.g., chemical shifts measurement in heteronuclear single quantum coherence [HSQC] spectra or relaxation rates) [23,38]. An interesting case involving (at least) three relevant conformational states is the allosteric PBX1 homeodomain (PBX-HD) [39,40]. This small DNA-binding protein comprises three α-helices that are packed against each other and an unstructured 15-residue extension at the C-terminus. DNA-binding is accompanied by folding of the C-terminal segment to a fourth helix, which enhances the binding of transcription factors to a remote site on the protein surface (Fig 1C). Elaborate RD NMR analysis showed that the disorder-to-helix transition of the C-terminal segment in PBX-HD already occurs, to a low extent (4.5 ± 0.8% at 25°C), in the absence of DNA at a rate *k*_ex_ of 2300 ± 200s^-1^. Folding of the C-terminal segment is accompanied by a concerted restructuring of the remainder of the protein, including residues that are involved in the recognition of transcription factors, which implies a functional role of these dynamics in the allosteric communication mechanism of PBX-HD. Valuable insights into the energetics of this process were derived from temperature-dependent RD measurements [39]. Moreover, combination of RD measurements with orthogonal NMR relaxation techniques revealed the presence of an additional high-energy state in the PBX-HD conformational ensemble, with an exchange rate, *k*_ex_, of 9,500 ± 2,300s^-1^, in which the C-terminal extension is locally misfolded [40]. The characterization of this state, which represents an off-pathway folding intermediate that corresponds to a kinetic dead end, highlights the complexity of the conformational ensemble that is present in solution.

## Magnetization Exchange Experiments

**Fig 3:**
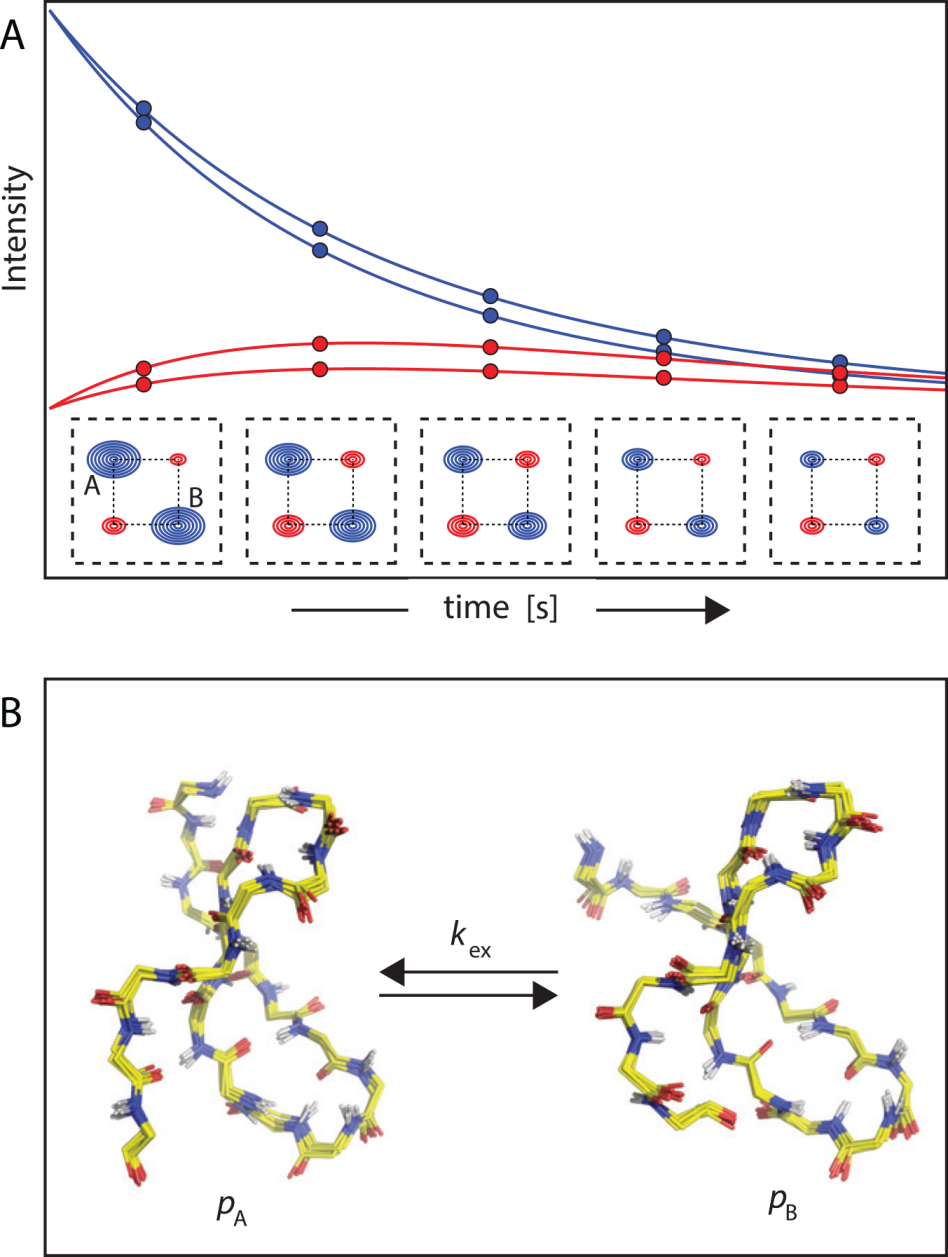
Magnetization exchange experiments. (A) In cases in which separate resonances are observed for states A and B, transitions between these states occurring in approximately hundreds of milliseconds can be monitored by magnetization exchange. In these experiments, exchange cross-peaks (shown in red) are observed that are directly related to the interconversion between A and B. (B) Analysis of peak intensities in magnetization exchange spectra with variable delay periods yields kinetic (*k*_ex_) and thermodynamic (*p*_A_, *p*_B_) information at multiple sites (e.g., NH groups) in proteins.

Conformational transitions can involve motions on even slower timescales, in which case NMR relaxation techniques that monitor magnetization exchange are suitable [41]. In these experiments, chemical shifts are recorded after a variable delay period, during which exchange between states occurs. During this delay, which is limited by the relaxation properties of the system, conformational states have a finite probability of converting to each other. The resultant spectrum thus contains cross-peaks that reflect the interconversion between states, along with diagonal peaks deriving from each conformer (Fig 3). By monitoring both the emergence of cross-peaks rate and the decay of diagonal peaks, the exchange kinetics between conformational states and, hence, their mean lifetimes, can be determined [10].

Methyl-TROSY-based magnetization exchange experiments have provided exciting insights into slow allosteric transitions of a number of high-molecular weight proteins and protein complexes [42,43]. For the archaeal 20*S* core-particle (CP) proteasome, analysis of methyl-TROSY magnetization exchange spectra revealed a delicate balance of different conformational states in solution that are critical for its function [44]. The 20*S* CP, a large protein complex that forms a barrel-like structure around a central proteolytic chamber (Fig 1D), maintains cellular homeostasis by selectively degrading misfolded or damaged proteins. Substrate entry is restricted by 13 Å gated pores at either side of the proteasome barrel that are regulated through binding of activators (regulatory particles). The magnetization exchange data showed that the gate, which is formed by N-terminal residues of the α-subunits in the 20*S* CP, exists in dynamic equilibrium between different states, one higher-populated ground state and two lower-populated states. It could further be shown that, in these less-populated conformers, the entrance to the proteolytic chamber is blocked, while in the ground state this is not the case. The NMR experimental data thus directly relate to the conversion of inactive states of 20*S* CP to its activated state, and vice versa. Of note, in X-ray crystallographic studies of the 20*S* CP, electron density has not been observed for the proteasome gating residues [45].

## NMR Order Parameters

**Fig 4:**
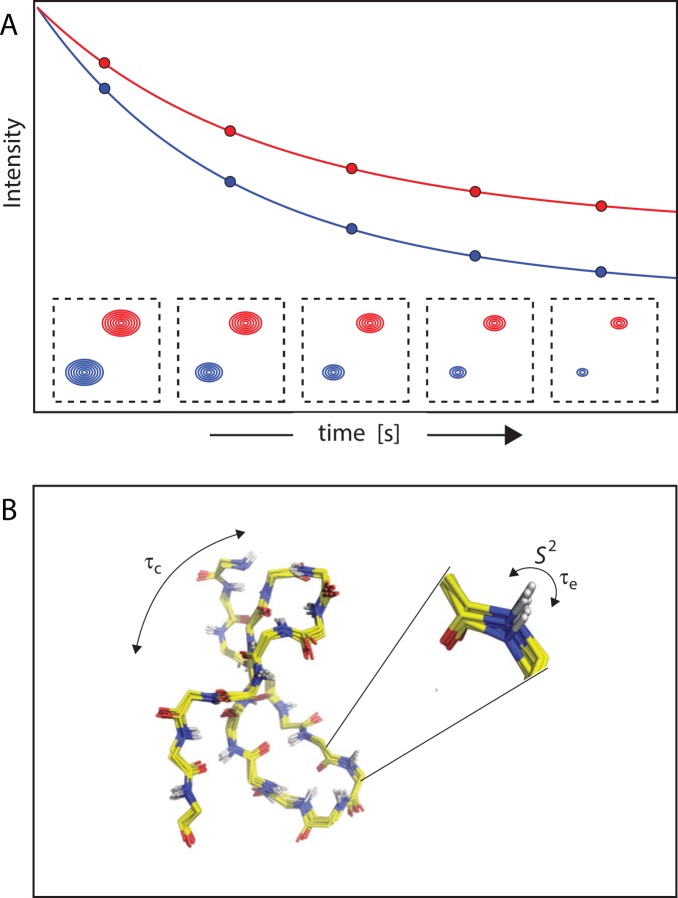
Determination of NMR order parameters. (A) Processes in the pico- to nanosecond time regime can be probed by experiments that monitor the relaxation rates of different spin modes. Relaxation rates at multiple sites in a protein are determined from exponential fits of resonance intensities in a time series. (B) Analysis of the experimental data within the model-free approach separates nanosecond timescale contributions arising from rotational diffusion of the protein as a whole (τ_c_) from (typically) picosecond contributions due to internal bond vector fluctuations, for which amplitudes (*S*^2^), timescale (τ_e_), and, if applicable, information on additional motions are obtained.

In addition to slow timescale motions, which frequently report on redistributions of states in conformational ensembles, allosteric proteins in many cases display functionally relevant pico-to-nanosecond dynamics. Motions in this time regime typically reflect stochastic fluctuations of bond vectors. Experimentally, such fast dynamic processes can be probed by NMR spin relaxation measurements that monitor different relaxation modes (Fig 4) [46,47]. The experimental data are typically interpreted within the "model-free" analytical strategy in terms of generalized squared order parameters *S*^2^ [48], which provide a generic measure of the amplitudes of bond vector fluctuations, along with the timescale (τ_e_) of these motions. As such, *S*^2^ reports on the degree of spatial restriction of internal motion of a particular bond vector. Values of *S*^2^ can vary from zero to one, with one corresponding to a rigid bond vector and zero corresponding to complete flexibility. Notably, the experimental relaxation data may require additional fitting parameters besides the standard *S*^2^ and τ_e_ pair, indicative of additional motions and/or micro- to millisecond dynamics [49]. While the "model-free" strategy has been dominating the analysis of pico- to nanosecond timescale dynamics, the use of specific motional models, such as the Gaussian axial fluctuation model [50], can be a practicable alternative for data interpretation, providing valuable information regarding the details of internal motions [51]. Moreover, the spectral density function that underlies NMR relaxation phenomena can be mapped at several frequencies [52].

Standard experimental setups typically involve the measurement of backbone amide NH bond vectors [53], but experiments for determining order parameters are also available for side-chain positions in amino acids, such as ^2^H nuclei in methyl groups of partly deuterated proteins [54]. Because ^13^C-^13^C scalar interactions in proteins or nucleic acids represent a major complication for the measurement of ^13^C relaxation in protein side-chains, site-specific labeling (reviewed in [46]) and/or experimental schemes for the suppression of ^13^C-^13^C scalar couplings are required [55]. Moreover, to provide an improved physical description of bond motions, it is possible to probe the relaxation parameters of multiple bond vectors of overlapping functional groups (such as backbone amide NH and ^13^Cα-^13^CO bond vectors) [47].

NMR order parameters have been used as surrogates for conformational entropy, as they provide a direct measure of a local increase or decrease of the flexibility of bond vectors, for example, upon binding of a ligand molecule [56,57]. In addition, because order parameters are sensitive to pico- to nanosecond timescale dynamics, such data presents an ideal interface between experimental techniques and molecular-dynamics-based approaches. Several studies are available in the literature that reproduce and, thereby, validate protein NMR order parameters from (sub-)microsecond MD trajectories [58]. Since computer simulations provide dynamic information of all bond vectors in a protein, not only those that are easily observable by NMR, the amount of information that is available for identifying correlated motions in proteins is significantly extended.

Most importantly, MD simulations complement NMR order parameters by providing collections of atomic coordinates that correctly represent the underlying dynamic processes. Given the nonlinear relationship between NMR relaxation and protein structure(s), however, a comprehensive representation of pico- to nanosecond dynamics data is not a trivial task. Not only are accurate, experimentally validated force fields essential and critical, but so is sufficient conformational sampling of the system. Sampling efficiency can be improved by use of enhanced sampling approaches such as replica exchange MD (REMD) [59] or accelerated MD (AMD) [60]. Quite generally, molecular dynamics studies have shown that structural fluctuations that are consistent with the experimental order parameters can indeed be reproduced by simulation [61]. The experimental data match the values that can be calculated from MD trajectories, demonstrating the synergistic potential of molecular dynamics and NMR.

NMR experimental data and molecular simulation have been integrated by using order parameters (along with structural information) as restraints in MD approaches to generate a structural ensemble of ubiquitin that adequately represents pico- to nanosecond timescale dynamics [62]. The obtained structures were cross-validated by back-calculating NMR observables that were not used for structure generation, such as scalar and residual dipolar couplings, showing high agreement. For ubiquitin, the dynamics-refined structural ensemble displays a significantly higher level of heterogeneity than ensembles obtained by standard NMR structure determination protocols. Most strikingly, integration of NMR and molecular simulation revealed a high degree of side-chain rotameric heterogeneity of hydrophobic residues that is not traceable by using standard NMR and X-ray structure determination protocols. It was hypothesized that the liquid-like mobility of the hydrophobic core in ubiquitin may well be a general feature of proteins that is essential for biological function [62].

With respect to allosteric proteins, NMR order parameters have revealed the dynamic nature of a number of different systems [63–65]. In CAP (Fig 1A), for example, differences of backbone amide NMR order parameters of different ligand complexes were used to estimate changes in conformational entropy upon cAMP binding [30]. Comparison with the calorimetrically measured difference in entropy between the two sequential cAMP binding steps confirmed that the observed negative cooperativity in this protein is indeed due to alterations in flexibility. Because ligand binding to CAP occurs without measurably changing the three-dimensional structure of this protein, CAP is considered a model system for purely dynamics-driven allostery. NMR order parameters have also revealed the dominating effect of conformational entropy in the allosteric activation of the DNA-binding domain by cAMP binding [66]. The CAP experimental data have been verified by extensive molecular dynamics simulations, further highlighting the entropic nature of allosteric coupling [67]. In a recent MD study, several side chain interactions that change the protein’s internal force network were identified, providing further insight into mechanistic details of the CAP allosteric mechanism [68]. Moreover, using force distribution analysis, a subset of protein sites in CAP could be identified that act as an allosteric communication pathway between the two cAMP and the DNA binding sites.

For the allosteric KIX domain of CBP (Fig 1B), backbone amide order parameters *S*^2^ indicate that binding of MLL peptide leads to an overall loss of pico- to nanosecond timescale dynamics in the KIX backbone [12]. Upon complex formation, MLL directly packs against the L_12_-G_2_ loop connecting helices α1 and α2, which is accompanied by a substantial rigidification of this particular region of KIX. In a molecular dynamics study, the mechanistic significance of the L_12_-G_2_ loop for allosteric communication KIX was first recognized [69]. It could be shown that rigidification of the L_12_-G_2_ loop acts as a dynamical switch in KIX that triggers allosteric communication between binding sites. In addition, MD simulations using a topology-based Go-like model showed that stabilization of the L_12_-G_2_ loop upon MLL binding to KIX reduces structural dynamics and lowers the entropic cost for binding the second ligand peptide at the remote allosteric site [70]. These data thus provide a causative link between changes in structural dynamics upon ligand binding and its effects on affinities in the KIX domain.

Experimentally, pico- to nanosecond timescale dynamic processes can also be probed by measuring NMR cross-correlated relaxation rates [71]. For high-molecular weight proteins, dipolar ^1^H-^1^H cross-correlated relaxation rates are directly related to order parameters and can thus be employed to characterize the amplitude of motion of a particular moiety. In case of methyl groups, *S*^2^_axis_ is obtained, i.e., the generalized order parameter that describes the amplitude of motion of the methyl 3-fold axis [72]. Intramethyl ^1^H-^1^H cross-correlated relaxation rates can be measured using highly deuterated, methyl-labeled samples, employing experiments that monitor the buildup of methyl ^1^H double- or triple-quantum magnetization [73,74].

For the allosteric enzyme imidazole glycerol phosphate synthase (IGPS**)**, dipolar ^1^H-^1^H cross-correlated relaxation rate constants, *η*, were determined for the apo-form of the enzyme, along with different binary and ternary complexes [75]. IGPS is a heterodimeric protein (Fig 1E) in which each monomer subunit catalyzes a different reaction. Effector binding to one subunit (HisF) accelerates the hydrolysis of glutamine by the other subunit (HisH) across a distance of >25 Å. ^1^H-^1^H cross-correlated relaxation rate constants for the different complexes indicated a site-specific response of pico- to nanosecond timescale motions upon binding of effector. In agreement with these data, a 100-nanosecond all-atom MD simulation revealed a variety of correlated motions on the nanosecond timescale, bridging the effector binding and active sites [76]. Detailed analysis of the MD trajectories using generalized correlation coefficients further showed that the entropically driven effector binding partly promotes structural changes in HisF and HisH that are responsible for the inactive-to-active allosteric transition of this enzyme. Because motions occurring on the millisecond timescale, detected by RD measurements, are required for optimal HisH activity, the authors hypothesized that these nanosecond dynamics might represent the initial fluctuations that ultimately lead to the formation of the fully active enzyme [75].

## Residual Dipolar Couplings and Chemical Shifts

The experiments described above directly probe dynamic processes, such as conformational exchange or bond vector fluctuations, through spin relaxation measurements. These methods are ideally complemented by NMR parameters that are dynamically averaged in conformational ensembles, such as chemical shifts or dipolar couplings. While these observables do not yield quantitative information on the timescales of dynamic processes per se, they can provide invaluable information about the dynamic nature of allosteric proteins and protein complexes.

**Fig 5:**
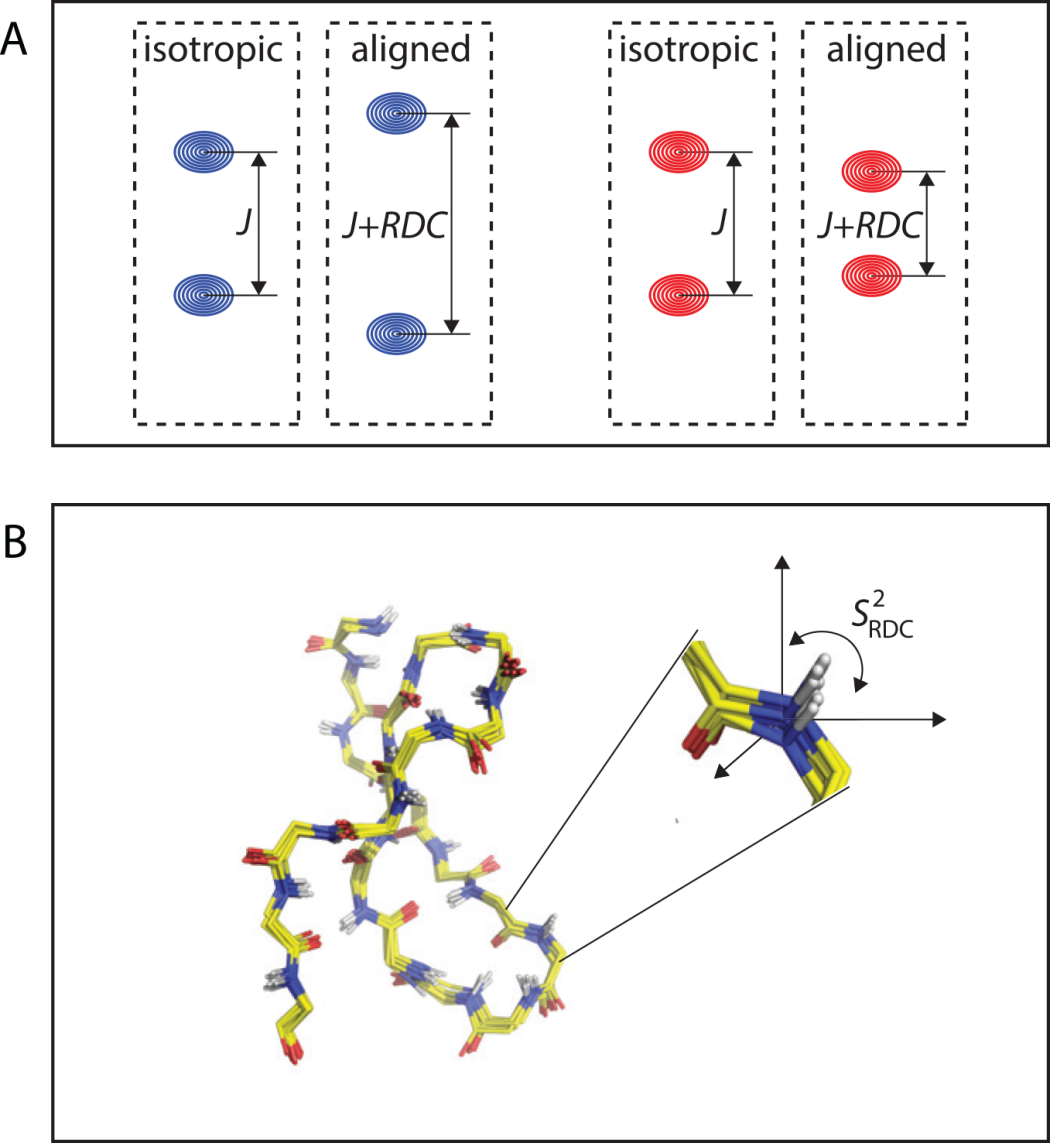
Dynamics from residual dipolar couplings (RDCs). (A) In isotropic solution, rotational diffusion averages dipolar couplings to zero and only scalar couplings *J* are observed. Weak molecular alignment of proteins impedes averaging of dipolar couplings to zero, and RDCs greater than or less than zero add to line splittings. (B) Residual dipolar couplings contain site-specific information on the orientation of internuclear vectors with respect to a molecular reference frame. Population-weighted averaged RDCs are observed if internal dynamics cause dipolar vectors to reorient. By combining experimental data from multiple molecular alignment media, structural and dynamic contributions can be separated to extract RDC-derived order parameters.

As an example, residual dipolar couplings (RDCs) represent a rich source of structural information and are routinely used for NMR structure determination, as they report on the relative orientation between vectors connecting pairs of interacting nuclei (e.g., the backbone NH bond vector) and the magnetic field [77]. In addition, in cases in which dynamics cause the dipolar vector to reorient, the magnitude of a given RDC value corresponds to the population-weighted averaged value over all orientations (Fig 5) [78]. This particular feature of RDCs provides the basis for their use as sensitive reporters on dynamic processes, spanning a broad time scale window that ranges from picoseconds all the way to milliseconds. In order to extract this information from the experimental data, however, both dynamical and structural parameters must be taken into account. Typically, RDCs are measured using multiple differently aligned samples and subsequently fit by use of explicit motional models, some of which yield per-residue generalized order parameters, or by generating ensembles of structures that adequately represent the experimental data [79]. Because dipolar coupling data by themselves do not report on timescales, this information is only indirectly available by comparison of RDC-derived order parameters with values obtained from spin relaxation data [79].

From an extensive set of RDC data that were recorded under variable experimental conditions, correlated dynamics in the immunoglobulin-binding B1 domain of streptococcal protein G were characterized [80]. A total of up to 27 RDCs were collected for the peptide planes of each amino acid and analyzed employing a three-dimensional Gaussian axial fluctuation model. The results indicate the presence of correlated motions amino acids across the central β-sheet. Based on these observations, it was concluded that long-range information transfer between remote parts of the structure is transmitted collectively through a network of residues that are connected via interstrand hydrogen bonds. Notably, RDC-derived order parameters were found to be consistently lower than the *S*^2^ values that were extracted from ^15^N relaxation experiments, implying that the correlated dynamics involve motions that are slower than pico- to nanoseconds.

As with NMR order parameters, molecular simulations have been employed to generate physically plausible structural ensembles that are consistent with the experimental RDC data. For ubiquitin, a 116-member structural ensemble was generated using a large RDC dataset as input restraints that covers solution dynamics up to the microsecond timescale [81]. The RDC ensemble embraces all structural heterogeneity that is observed in crystal structures of ubiquitin and ubiquitin complexes, indicating that microsecond and submicrosecond dynamics account for a large proportion of the structural variability that is required for molecular recognition. While RDC ensembles of allosteric proteins are not available to date, it is clear that this approach has the potential to substantially improve our understanding of allosteric mechanisms.

NMR chemical shifts, like residual dipolar couplings, are exquisitely sensitive to even small changes in structure. Because NMR chemical shifts observed for each peak correspond to an average over the chemical shifts in each conformer in the ensemble, analysis of peak positions in NMR spectra yields quantitative information on the populations of different conformational states. Moreover, chemical shifts contain information about dynamics, since conformational averaging affects the experimentally observed values. Significant deviation of NMR chemical shifts from random-coil values thus indicate a relatively rigid and well-defined structure, while proximity of chemical shifts to random coil values is a manifestation of conformational averaging. By use of empirically derived relationships between chemical shifts and flexibility, this feature can be employed to predict dynamic properties at protein backbone and side-chain sites using chemical shift data [82,83]. The timescale of dynamic processes that is covered by this method ranges from picoseconds to milliseconds [84].

With respect to dynamic allostery, NMR chemical shift measurements have predominantly been employed for analyzing the population shifts of conformational states [85–87]. A particularly powerful experimental probe is methyl carbon ^13^C chemical shifts of isoleucine, leucine, and valine, which provide a very sensitive measure of the side-chain conformations of these residues [88]. Assuming that conformational sampling of side-chain dihedral angles (χ2 in leucine and isoleucine, χ1 in valine) can be described in terms of jumps between a limited number of rotameric states, experimentally determined methyl ^13^C chemical shifts of these residues can be used to determine the populations of these rotameric states [89–91]. This particular feature was used to characterize allosteric coupling in the KIX domain [12]. Methyl ^13^C chemical shifts were used to analyze the isoleucine, leucine, and valine side chain rotameric states in different complexes of KIX. The data revealed that the transition from the binary complex of KIX (with the MLL peptide bound) to the ternary complex (with both MLL and pKID peptides bound) (Fig 1B) is accompanied by a collective change of KIX side-chain rotameric states in the hydrophobic core. The redistribution of rotameric states involves residues that had previously been recognized as being part of the allosteric network [34]. Notably, this effect is not captured by the NMR structural bundles of KIX complexes, which represent the highest-populated rotamers of these residues in solution. Using metadynamics, the allosteric communication pathways of KIX were probed computationally, revealing tight coupling of the dynamics of the L_12_-G_2_ loop connecting helices α1 and α2 and the restructuring of the hydrophobic core [35]. It was concluded that binding of the MLL to KIX moves the L_12_ loop close to the ligand peptide, which results in a less compressed hydrophobic core that is susceptible to a redistribution of rotameric states of allosteric network residues. Ultimately, this process leads to an increase of the binding affinity for the second ligand.

Of specific interest are side-chain methyl-TROSY experiments [92], which have been applied to monitor population shifts for very high-molecular weight allosteric proteins up to 670 kDa [93–95]. For example, it could be demonstrated that activator binding to 20*S* CP (Fig 1D) modulates the relative populations of conformational states that are present in solution [96]. Moreover, the methyl-TROSY ^13^C-^1^H chemical shift data revealed that the activator binding site is linked to the active site of the 20*S* CP through an allosteric network spanning a distance of 80 Å. It was further shown that the function of the 20*S* CP is perturbed by changing the relative distribution of conformers. Binding of an allosteric inhibitor, which shifts the position of the equilibrium to the inhibited state, was monitored by methyl ^1^H and ^13^C chemical shifts and resulted in a loss of function. In addition, population shifts were induced by mutation of amino acid residues that make key contacts with allosteric activators, resulting in an approximately linear dependence of chemical shifts observed in methyl-TROSY spectra [96].

While these examples do not exploit NMR chemical shifts as reporters for dynamics, they clearly demonstrate the dynamic nature of conformational ensembles of proteins in solution. Indeed, chemical shift responses to perturbations are very commonly employed as diagnostic tools for identifying coupled networks within allosteric proteins or protein complexes. This task becomes increasingly challenging, however, in cases in which allosteric proteins lack significant long-range structural responses to perturbations, indicative of fundamentally dynamics-driven allostery [97]. In such cases, covariance analysis of chemical shift changes caused by binding different effector molecules can be employed [98]. Using this approach, protein sites with large chemical shift variations upon perturbation as well as those that display only subtle variations are taken into account, so long as their chemical shift responses are correlated. Covariance analysis of chemical shifts is, thus, capable of identifying networks of coupled residues that comprise both structural and dynamic components. As a matter of fact, this approach is particularly effective in identifying allosteric coupling networks within partially unstructured and highly dynamic regions, which are common in proteins that are involved in signaling and often remain elusive to structure-based techniques [99]. Chemical shift-based perturbation analysis of allosteric networks is complemented by computational approaches using elastic network models. Scanning for protein sites that dynamically respond to perturbations of ENMs, such as ligand binding, can identify coupled networks within a protein that are related to allosteric communication [100].

## Outlook

The above examples show that allosteric communication in many cases includes structural transitions, accomplished through a defined and dynamic redistribution of the conformational ensemble. As proposed by Cooper [101], allostery can in principle be completely dynamic in nature without requiring any structural changes at all. The question thus remains: how small a structural change shall be considered significant? From a practical perspective, this represents a challenge for experimental techniques that are exquisitely sensitive to structural changes. Crystallographic studies of proteins have proven the exclusive ability of high-resolution X-ray diffraction techniques to detect even small differences between three-dimensional structures. Likewise, methodological advancements in NMR spectroscopy have enabled the observation of conformational states in solution that are only sparsely populated as well as transitions between them.

Future developments in this direction, along with the enhanced spectrometer performance that is afforded by cryogenically cooled probes and higher magnetic field strength spectrometers, will further boost the sensitivity of NMR experiments. This will enable NMR studies of even higher-molecular-weight allosteric proteins and protein complexes. Slow conformational transitions in such systems are likely outside the time regime that can be monitored by current magnetization transfer techniques, requiring the development of novel experimental approaches such as the use of long-lived spin states for detecting very slow exchange processes [102]. Of note, data acquisition of two-dimensional methyl-TROSY spectra in less than 5 seconds has been reported [103], providing an experimental tool for real-time observation of conformational transitions in higher-molecular-weight assemblies. NMR real-time approaches to characterize slow conformational transitions have been particularly useful for medium-sized ribonucleic acids in the past [104]. A number of allosteric ribozymes and riboswitch aptamers have been described, in which remote parts of the RNA are likely dynamically coupled [105,106].

Moreover, it is foreseeable that even lower-populated states will become amenable to experimental characterization, further increasing the proportion of the conformational ensemble that can be observed by experiment. Recent crystallographic studies have highlighted the capability of room temperature X-ray data collection to model multiple conformers in crystals [107]. These techniques thus offer intriguing synergistic potential for NMR spectroscopy and X-ray crystallography to characterize conformational heterogeneity at great detail by use of orthogonal approaches. Distinctive structural features that are difficult to grasp with standard structure determination protocols, such a side-chain rotameric distributions, are likely to attract considerable attention in the future. It is tempting to speculate that ligand-induced modulation of side-chain heterogeneity, as observed for the KIX domain, may represent a common aspect of allosteric communication. Taken together, experimental advances will present new challenges for computational techniques with respect to both the timescale that is accessible by computer simulations as well as the size of the biomolecules that are investigated. All-atom metadynamics [108] and AMD [109] represent but two approaches that are available to date for sampling millisecond timescale events, setting the stage for future developments toward even slower timescale motions. Only a combination of experimental and computational techniques will succeed in providing a comprehensive and authentic view of dynamic allostery in biomolecules.
